# Transcriptome research of human amniocytes identifies hub genes associated with developmental dysplasia in down syndrome

**DOI:** 10.18632/aging.205291

**Published:** 2023-12-12

**Authors:** Zhenglong Guo, Hai Xiao, Wenke Yang, Tao Li, Bingtao Hao, Shixiu Liao

**Affiliations:** 1 Henan Provincial Key Laboratory of Genetic Diseases and Functional Genomics, National Health Commission Key Laboratory of Birth Defects Prevention, Medical Genetic Institute of Henan Province, Henan Provincial People’s Hospital, People’s Hospital of Zhengzhou University, Zhengzhou, China; 2 School of Medicine, People’s Hospital of Henan University, Henan University, Zhengzhou, China; 3 Cancer Research Institute, School of Basic Medical Sciences, Southern Medical University, Guangzhou, China; 4 Department of Medicine Laboratory, Fuwai Central China Cardiovascular Hospital, Zhengzhou, China

**Keywords:** RNA sequencing, HSA21, Down syndrome, developmental disorder, amniocytes

## Abstract

Trisomy 21, or Down syndrome (DS), is the most frequent human autosomal chromosome aneuploidy, which leads to multiple developmental disorders, especially mental retardation in individuals. The presence of an additional human chromosome 21 (HSA21) could account for the pathological manifestations in DS. In this study, we analyzed the mRNA gene expression profile of DS-derived amniocytes compared with normal amniocytes, aiming to evaluate the relationship between candidate dysregulated HSA21 genes and DS developmental phenotypes. Differentially expressed genes (DEGs) included 1794 upregulated genes and 1411 downregulated genes, which are mainly involved in cell adhesion, inflammation, cell proliferation and thus may play an important role in inducing multiple dysplasia during DS fetal development. Furthermore, STRING protein network studies demonstrated 7 candidate HSA21 genes participated Gene Ontology (GO) terms: cell adhesion and extracellular matrix remodeling (*COL6A1*, *COL6A2*, *COL18A1*, *ADAMTS5*, *JAM2*, and *POFUT2*), inflammation and virus infection response (*MX1* and *MX2*), histone modification and chromatin remodeling (*NRIP1*), glycerolipid and glycerophospholipid metabolism (*AGPAT3*), mitochondrial function (*ATP5PF* and *ATP5PO*), synaptic vesicle endocytosis (*ITSN1* and *SYNJ1*) and amyloid metabolism (*APP*). Meanwhile, GSEA enrichment identified several transcription factors and miRNAs, which may target gene expression in the DS group. Our study established connections between dysregulated genes, especially HSA21 genes, and DS-associated phenotypes. The alteration of multiple pathways and biological processes may contribute to DS developmental disorders, providing potential pathogenesis and therapeutic targets for DS.

## INTRODUCTION

Down syndrome (DS), also named trisomy 21 (T21), is one of the most common human genetic disorders, manifested by multiple clinical features, including intellectual disability, increased risk of cardiovascular and autoimmune diseases, susceptibility to infections and other congenital malformations, affecting approximately 1 in 650-1000 livebirths worldwide [1–3]. The presence of an extra copy of human chromosome 21 (Chr21/HSA21) could account for DS pathological phenotypes, despite that HSA21 is the smallest chromosome and constitutes only approximately 1% of the human genome. Advanced maternal age is closely related to segregation errors in HSA21 during meiotic processes and is thus regarded as the major risk factor for DS occurrence [4]. Regarding the pathogenic mechanism in DS, the most widely accepted hypothesis is a gene dosage effect resulting from the extra HSA21, in which a 1.5-fold change in gene expression would alter specific cellular processes or pathways. For example, the amyloid-beta precursor protein (*APP*) gene-encoded amyloid protein level is associated with early-onset Alzheimer’s disease (AD), which has been characterized in DS patients [5]. Down Syndrome Critical Region Gene 1 (*DSCR1*) and Dual Specificity Tyrosine Phosphorylation Regulated Kinase 1A (*DYRK1A*) are reported as regulators of vertebrate development through modulating Nuclear Factor of Activated T Cells (NFAT) transcriptional activity [6]. High Mobility Group Nucleosome Binding Domain 1 (*HMGN1*) plays an important role in leukemia progression observed in DS [7, 8]. Synaptojanin 1 (*SYNJ1*) contributes to DS-related brain dysfunction in Ts65Dn mice [9]. Another hypothesis proposed from some studies is that aneuploidies, including trisomy 21, 18 and 13, share similar phenotypes, such as decreased cell proliferation ability, suggesting that aneuploidy drives the DS phenotype independent of triplicated gene identity [10, 11].

To explore the molecular mechanism of HSA21 genes leading to DS phenotypes, several cell models derived from DS patients have been established for global gene expression profiling studies, including blood cells [12], induced pluripotent stem cells (iPSCs) [13, 14], primary fibroblasts [15, 16], monocytes, T cells and B cells [17]. Due to the expression difference between the adult and embryonic stages, conclusions from these studies may not represent fetal development in DS. Human amniotic fluid (AF), which is always collected from the second trimester pregnant woman, is a routine method of prenatal diagnosis, especially for aneuploids [18]. Although amniocytes in AF contain heterogeneous cell types, these cells derived from the fetus could be more suitable for monitoring the cellular stage of fetal development [19].

In this study, we aimed to analyze the mRNA gene expression profile of amniocytes from normal and DS amniocytes, including male and female samples, through RNA-seq. Considering the potential function of HSA21 genes in regulating the DS phenotypes, we combined the screening of differentially expressed genes with functional enrichment including cellular process and pathway, together with an interactive network assay. These data established a reference resource for further studies on candidate HSA21 genes in regulating DS fetal development and therefore provide potential therapeutic targets.

## MATERIALS AND METHODS

### Amniocyte culture and karyotyping

This study was conducted in the Prenatal Diagnosis Center of Henan Provincial People’s Hospital (Zhengzhou, China). Pregnant women came for further prenatal diagnosis due to high risk of Down syndrome during the second trimester screening. After genetic counseling, ultrasound and serological examination, amniocentesis (AC) was applied to acquire 16 ml amniotic fluid. The amniotic fluid was divided equally into two parts, centrifuged at 1200 rpm for 10 min, and seeded into two 25 cm^2^ culture flasks under sterile conditions. Amniocytes were cultured in BIO-AMF™-3 Medium (Biological Industries, Israel) and maintained at 37°C in a 5% CO2 incubator with the medium changed at day 8. At day 11, half of the cultured cells were used for further karyotyping. Briefly, amniocytes were incubated with 2 μg/ml colchicine (Sigma, USA) in fresh culture medium for 6 h to enrich arrested metaphases, followed by digestion and treatment with 0.75 M KCl for 40 min at 37°C, fixed with cold methanol:acetic acid (3:1) 3 times and spread onto a precooled clean glass slide. For G-band analysis, cells were digested with 0.25% trypsin and stained with Giemsa solution, followed by analysis with standard protocols. At least 10 individual cells were scanned and analyzed with a high-throughput fully automatic chromosome scanning platform (Leica, Wetzlar, Germany).

### RNA sequencing

After karyotype diagnosis, residual cells were harvested for RNA sequencing. Total RNA was extracted from DS (10 samples) and normal amniocytes (5 samples) using TRIzol (Invitrogen, USA) according to the manufacturer’s instructions. Subsequently, total RNA was quantified using an Agilent 2100 bioanalyzer (Thermo Fisher Scientific, USA) and purified using oligo(dT)-attached magnetic beads. The library was constructed, and single-end 50-base reads were generated on the MGI2000 platform (BGI, Shenzhen, China). Raw data were filtered with SOAPnuke to remove adaptor sequences, unknown reads containing polyN sequences, and low-quality reads, thus obtaining clean reads and stored in FASTQ format. The clean reads were mapped to the Homo sapiens (GRCh38) reference genome using HISAT2/Bowtie2. R (version 3.5) was used to obtain CPM (counts per million) of mRNA expression and further principal component analysis and heatmap. Differentially expressed genes (DEGs) were identified by setting padj (*Q* value) < 0.05 and |log2 (fold change)|≥0.59 using DESeq2. The correlation heatmap, mRNA expression distribution, Gene Ontology (GO), Kyoto Encyclopedia of Genes and Genomes (KEGG) and Gene Set Enrichment Analysis (GSEA) were performed on the online Dr. Tom software (https://biosys.bgi.com) with *p* adj (*Q* value) < 0.05 as the threshold. To quantify the relative CPM expression per gene, the average CPM per gene was calculated based on the ratio of the CPM sum of all genes for a given chromosome to the gene number for the corresponding chromosome.

### Protein-protein interaction analysis

To explore the interactions of DEGs, the online interaction database platform STRING v.11.0 (https://string-db.org/) was applied in our study. Briefly, upregulated and downregulated genes were searched in “Multiple Proteins by Names/Identifiers” to build the protein–protein interactive (PPI) network. “Full STRING network type”, “highest confidence (0.900) interaction score”, “interactive svg display mode”, and “hide disconnected nodes in the network” were chosen to retain the interacting proteins with high confidence. FDR<0.05 was considered statistically significant to perform functional enrichments of GO of biological process and KEGG pathway. To determine the function of HSA21 genes in the PPI network, dysregulated HSA21 genes were selected in the network to re-center network on this node.

### Quantitative real-time PCR (RT-qPCR)

Total RNA of amniocytes from different group was extracted using TRIzol (Invitrogen, USA) according to the manufacturer’s instructions. Nanodrop (Thermo Fisher Scientific, USA) was applied to determine the quality and concentration of RNA, and RevertAid RT kit (Thermo Fisher Scientific, USA) was used to prepare cDNA. Real-time PCR was performed on Applied Biosystems StepOnePlus qPCR system with SYBR qPCR Master Mix (Q712, Vazyme, Nanjing, China). 2^−ΔΔCt^ method was used to quantify relative mRNA expression through actin beta (ACTB) normalization, in which the primers used were listed in Supplementary Table 1.

### Cell proliferation assay

Cell proliferation of amniocytes at passage 2 from normal and DS group were assessed using the Cell-Light EdU DNA Cell Proliferation Kit (RiboBio, Guangzhou, China) according to the manufacturer’s instructions. The proliferation rate was calculated using the ratio of the number of EdU-positive cells to the number of Hoechst 33342-positive cells, with three replicates for each group.

### Availability of data and materials

The clean data in FASTQ format of this study have been deposited into CNGB Sequence Archive (CNSA) [20] of China National GeneBank DataBase (CNGBdb) [21] with accession number CNP0003748.

## RESULTS

### RNA-sequencing results

**Figure 1:**
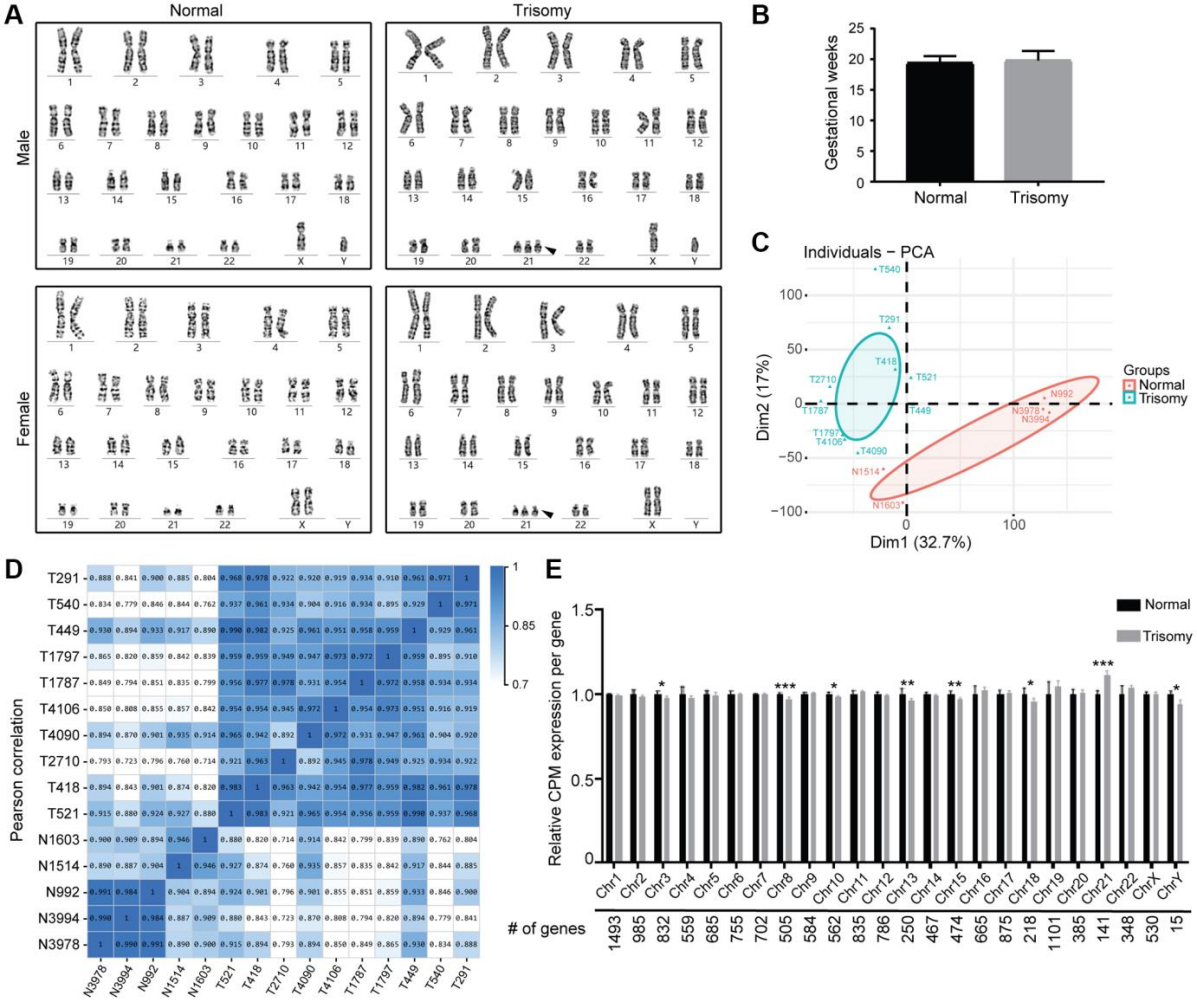
**RNA sequencing of amniocytes derived from the normal and trisomy 21 groups.** (**A**) Karyotyping results of cultured amniocytes from the trisomy 21 and normal groups, including males and females. Black arrow indicates the additional Chr21. (**B**) Gestational age of pregnant women in this study (nNormal = 5, nTrisomy = 10). (**C**) PCA plot comparing the normal and trisomy 21 groups according to their mRNA expression using counts per million (CPM) normalization. (**D**) Pearson correction heatmap of different samples in the normal and trisomy 21 groups. (**E**) Average mRNA expression per chromosome was calculated and quantified in the normal and trisomy 21 groups (^*^p < 0.05, ^**^p < 0.01, ^***^p < 0.001, two-tailed *t* test).

Through karyotype diagnosis, DS amniocytes could be identified by the observed additional HSA21 with the analysis result of 47, XX/XY, +21 (Figure 1A). In this study, we generated single-end RNA-seq data from 15 cell samples, including 10 DS samples (5 male, 5 female) and 5 normal samples (3 male, 2 female). After filtering adaptor sequences, unknown reads, and low-quality reads, approximately 21–24 million clean reads were mapped to the Homo sapiens (GRCh38) reference genome. In total, approximately 94% of the reads could be mapped, of which more than 80% were uniquely mapped to the human genome (Supplementary Table 2). Considering the uncertain effects of developmental stages on the gene expression profile, the amniocytes were all derived from the second-trimester pregnant women with gestational weeks 17–23 (Figure 1B).

We used CPM normalization to remove low-expressed genes in R, thus obtaining 14,742 genes for further studies, in which the distribution of CPM expression was relatively uniform (Supplementary Figure 1A). Principal component analysis (PCA) plot and Pearson correlation heatmap results demonstrated two separate clusters named the Normal group and Trisomy group, respectively, in which the included samples showed stable repeatability and correlation (Figure 1C, 1D). Meanwhile, the quantification data of mRNA gene expression in different chromosomes manifested significant upregulation in Chr21 as expected, as well as apparent downregulation in chr8, chr13, chr15, chr3, chr10, chr18 and chrY, indicating changes in the stability of the whole chromosome genome that result from trisomy 21 (Figure 1E).

### Differential gene expression analysis and functional enrichment

**Figure 2:**
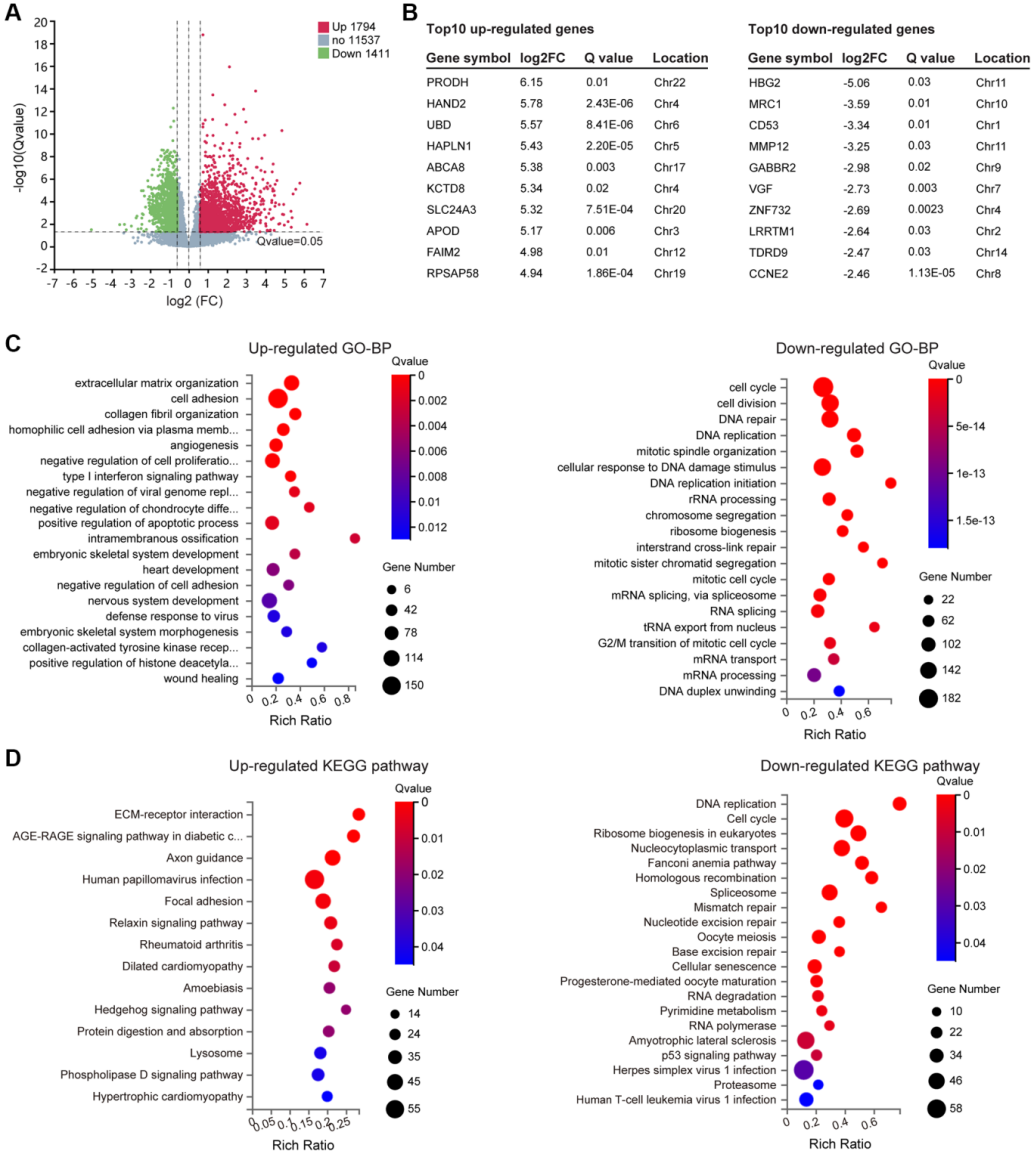
**Differentially expressed genes (DEGs) screening and enrichment analysis.** (**A**) Volcano plot of all expressed genes in Normal versus Trisomy. DEGs were filtered through the Fold change ≥1.5 and *Q* value ≤ 0.05. Dots represent individual genes, with upregulated genes in red, downregulated genes in green and others in gray. (**B**) List of the top 10 up- and downregulated genes in the DEG gene set. (**C**) GO enrichment of biological processes (BP) analysis of annotated DEGs; the top 20 are listed. (**D**) KEGG pathway enrichment analysis of annotated DEGs.

In DS, three copies of HSA21 should result in a 1.5-fold change in gene expression, therefore, differentially expressed genes (DEGs) were ultimately identified by setting padj (*Q* value) <0.05 and |log2 (fold change)|≥0.59 through DESeq2. In total, 3205 DEGs containing 1794 upregulated genes and 1411 downregulated genes were selected (Figure 2A, Supplementary Table 3), and the information of the top 10 DEGs is listed separately (Figure 2B). To gain insight into the phenotypic differences in trisomy 21 versus normal, we performed Gene Ontology (GO, (http://www.geneontology.org/)), Kyoto Encyclopedia of Genes and Genomes (KEGG, (https://www.kegg.jp/)) and Gene Set Enrichment Analysis (GSEA, (https://www.gsea-msigdb.org/)). The results are shown as follows.

#### GO enrichment

GO enrichment of biological processes (GO-BP) was carried out for functional classification of the annotated DEG gene set. Multiple GO terms were enriched, including 47 upregulated terms and 246 downregulated terms (Supplementary Table 4), in which top 20 were listed (Figure 2C). Among them, the upregulated genes were most closely related to “Cellular component organization and biological adhesion”. In addition to the top 4 listed terms including extracellular matrix organization, cell adhesion, collagen fibril organization and homophilic cell adhesion via plasma membrane adhesion molecules, some other GO terms were also identified, such as negative regulation of cell adhesion, axon guidance, cell-matrix adhesion, cell-cell adhesion, elastic fiber assembly and positive regulation of epithelial to mesenchymal transition. In addition, the upregulated GO terms also referred to “Developmental process”, mainly relevant to angiogenesis, embryonic skeletal system development, heart development, nervous system development, embryonic skeletal system morphogenesis, muscle structure development, odontogenesis and face morphogenesis. Moreover, “Immune system process and regulation” was likewise involved, for instance, type I interferon signaling pathway, negative regulation of viral genome replication, defense response to virus, positive regulation of interleukin-13 production, immune system process and response to virus. It is worth mentioning that synaptic vesicle exocytosis, synapse organization and endocytosis could directly affect the “nervous system or nerve cell function”. Eventually, “mitochondria function” (regulation of protein targeting to mitochondrion) and “chromatin remodeling and epigenetic regulation” (positive regulation of histone deacetylation) may play an important role in the DS phenotype as well.

The GO-BP terms enriched from downregulated genes were relatively consistent, principally involving in “Cell division and DNA replication” process, not only in mitosis but also in meiosis. Meanwhile, changes occurred in “DNA repair”, “Ribosome function”, “RNA splicing” and “RNA metabolism”, which probably represent the poor cellular state of DS-derived amniocytes.

#### KEGG enrichment

To further illustrate the pathways affected by the DEG gene set, up- and downregulated KEGG pathways were analyzed. Corresponding to the GO enrichment results, upregulated genes mostly activate “Cytoskeleton and adhesion” associated pathways, including ECM-receptor interaction, Axon guidance and Focal adhesion. Additionally, some disease-causing pathways were enriched, which could be divided into three parts: I) “Infectious/immune disease” (Human papillomavirus infection; Amoebiasis; Rheumatoid arthritis); II) “Cardiovascular disease” (Dilated cardiomyopathy; Hypertrophic cardiomyopathy); III) “Endocrine and metabolic disease” (AGE-RAGE signaling pathway in diabetic complications). Besides, “Signal transduction pathways” (Relaxin signaling pathway, Hedgehog signaling pathway, Phospholipase D signaling pathway), “Lysosome” and “Protein digestion and absorption” were also involved (Figure 2D).

In accordance with the downregulated GO-BP terms described above, “Cell growth, replication and repair” was the topical subject of the downregulated KEGG, which is displayed in Figure 2D. In addition, we found “neurodegenerative disease” (amyotrophic lateral sclerosis), which correlated with DS mental retardation, and “infectious disease” (herpes simplex virus 1 infection, human T-cell leukemia virus 1 infection) were also presented in the list.

#### GSEA analysis

Meanwhile, we utilized GSEA to explore the alteration of whole gene expression in specific gene sets, which could accurately reflect changes related to phenotypes in trisomy 21 versus normal tissues. First, the “KEGG subset of canonical pathways”, which belonged to the C2: curated gene sets, was selected for screening candidate pathways contributing to phenotypes in the trisomy/DS group. Compared with the normal group, “ECM-receptor interaction”, “Focal adhesion” and “Axon guidance” were the top 3 positively associated pathways in the trisomy 21 group, coinciding with the activated GO and KEGG results. “Hypertrophic cardiomyopathy (HCM)” and “arrhythmogenic right ventricular cardiomyopathy (AVRC)” were in agreement with “cardiovascular disease” related KEGG pathways and “heart development” related GO-BP terms. In addition, “leukocyte transendothelial migration” and “cytokine-cytokine receptor interaction” may refer to immune system function. The “Hedgehog signaling pathway” and “VEGF signaling pathway” also showed obvious correlations with the trisomy 21 group (Supplementary Figure 2A).

Of all the negatively correlated pathways in the trisomy 21 group, “DNA replication, DNA repair and cell cycle”, “RNA splicing and metabolism” and “oocyte meiosis and maturation” were the core suppressed pathways (Supplementary Figure 2B). Finally, we applied the “C3: regulatory target gene sets” collection to call regulatory motifs that could function as potential transcription factors and microRNA targets. As expected, multiple transcription factors, including vitamin D receptor (VDR), zic family member 3 (ZIC3), sterol regulatory element binding transcription factor 1 (SREBF1/SREBP1), general transcription factor IIi (GTF2I/TFIII), nuclear factor kappa B subunit 1 (NFKB1/NFKB), replication initiator 1 (REPIN1/AP4), sp1 transcription factor (SP1), POZ/BTB and AT hook containing zinc finger 1 (PATZ1/MAZR), visual system homeobox 2 (VSX2/CHX10), and LIM homeobox 3 (LHX3), may work as positive regulators in the trisomy 21 group. In contrast, the E2F transcription factor family (E2F), which mainly targets DNA replication and the cell cycle, should act as negative regulators in the trisomy 21 group (Supplementary Table 5). Some regulatory miRNAs might possess regulatory abilities in gene expression in the trisomy group, such as miR-518c, miR-331 and miR-296 (Supplementary Table 6).

### Changes in gene expression on HSA21

**Figure 3:**
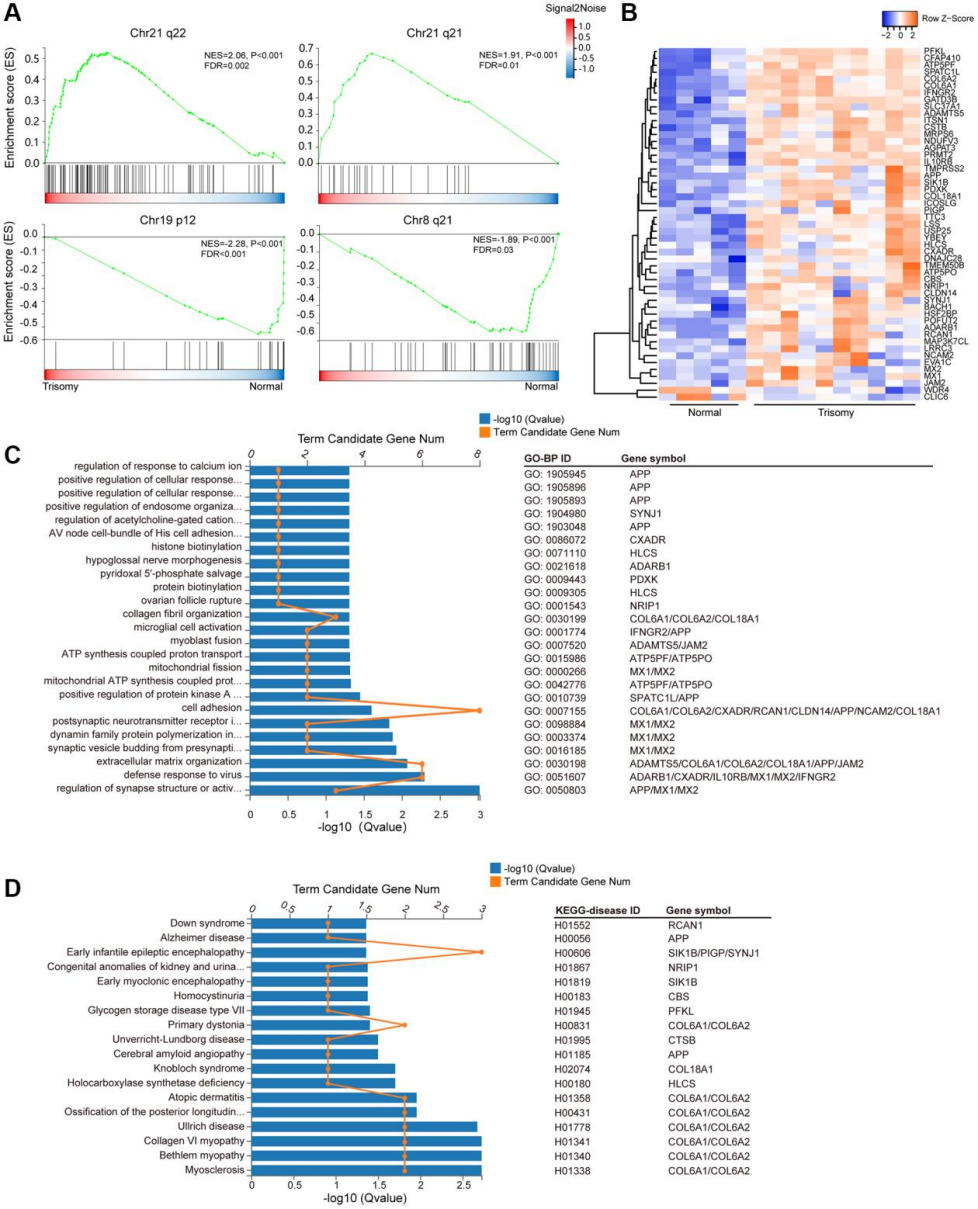
**HSA21-related DEG enrichment.** (**A**) Gene set enrichment analysis (GSEA) of all expressed genes in the “C1:positional” gene sets collection of MSigDB (Broad Institute). Abbreviations: ES: enrichment score; FDR: false discovery rate (adjusted *p* value). (**B**) Heatmap of the expression level of the DEGs on HSA21 (blue, low; red, high expression). (**C**) Enriched GO-BP terms and involved genes of annotated DEGs on HSA21. (**D**) KEGG disease enrichment analysis of annotated DEGs on HSA21.

Given that additional HSA21 in DS could most likely lead to the difference of gene expression on HSA21, therefore, we compared the expression levels of all 141 genes on HSA21 in the trisomy 21 versus normal groups, from which we observed a more obvious upregulation of mRNA gene expression on HSA21 (median of log2FC = 0.53) than on all genes (median of log2FC = 0.05) (Supplementary Figure 1B, 1C). Moreover, we used the “C1:positional” gene set collection of MsigDB, which was derived from the Chromosome and Karyotype band tracks from Ensembl BioMart, to reflect chromosomal deletions or amplifications and dosage compensation. As expected, activated genes in the trisomy 21 group were mostly situated on q21-22 of Chr21, indicating dosage compensation of HSA21-related genes. In addition, suppressed genes were also enriched on some other chromosomes, such as chr19 p12 and chr8 q21, indicating genomic instability in the trisomy 21 group (Figure 3A).

Meanwhile, we individually selected HSA21 genes from the DEG gene set. In total, 51 DEGs were located on HSA21, including 49 upregulated genes and 2 downregulated genes (Figure 3B, Supplementary Table 7). Afterwards, GO-BP and KEGG-disease enrichment were analyzed to classify the gene function and involved disease, in which some similar GO terms enriched from the whole DEGs were also presented. For instance, *COL6A1*, *COL6A2*, *COL18A1*, *ADAMTS5*, *JAM2*, *APP*, *CXADR*, *RCAN1*, *CLDN14* and *NCAM2* participate in “extracellular matrix organization”, “cell adhesion” and “collagen fibril organization”; *ATP5PF* and *ATP5PO* are related to “ATP synthesis”; and *MX1*, *MX2*, *IL10RB*, *IFNGR2*, *ADARB1* and *CXADR* are involved in the “defense response to virus”. In addition, some genes (*MX1*, *MX2*, *APP*, and *ADARB1*) could directly cause nerve system dysfunction through “Regulation of synapse structure or activity”, “Synaptic vesicle budding from presynaptic endocytic zone membrane”, “Postsynaptic neurotransmitter receptor internalization” and “Postsynaptic neurotransmitter receptor internalization” processes (Figure 3C). Based on the vital function of the HSA21 DEGs mentioned in the KEGG DISEASE database, these genes (*COL6A1*, *COL6A2*, *COL18A1*, *APP*, *RCN1* and others) were closely related to some congenital malformations or genetic diseases, mainly for muscle and nervous system diseases (Myosclerosis, Alzheimer disease, Down syndrome, and others) (Figure 3D).

### STRING protein network analysis of DEGs

**Figure 4:**
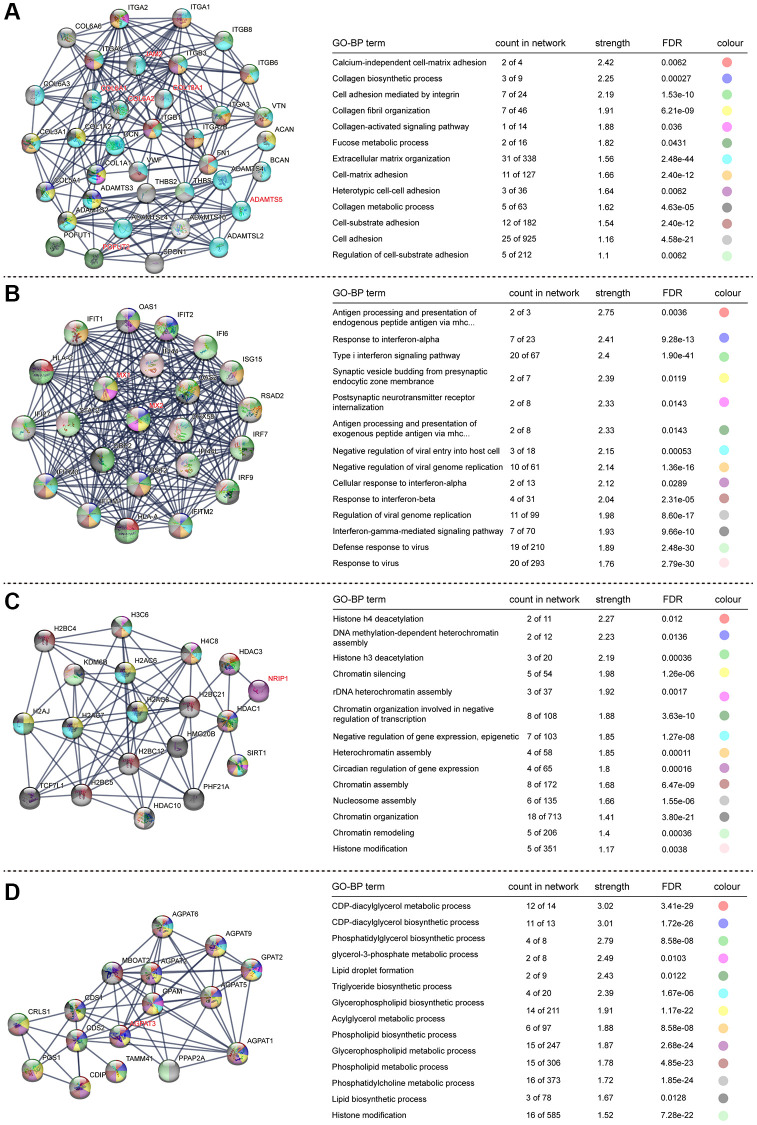
**STRING protein network analysis of upregulated genes.** (**A**–**D**) Different networks clustered in the upregulated genes. Dots indicate interacting proteins, and colours denote GO-BP terms enriched in each network. The HSA21-related genes are marked in red.

**Figure 5:**
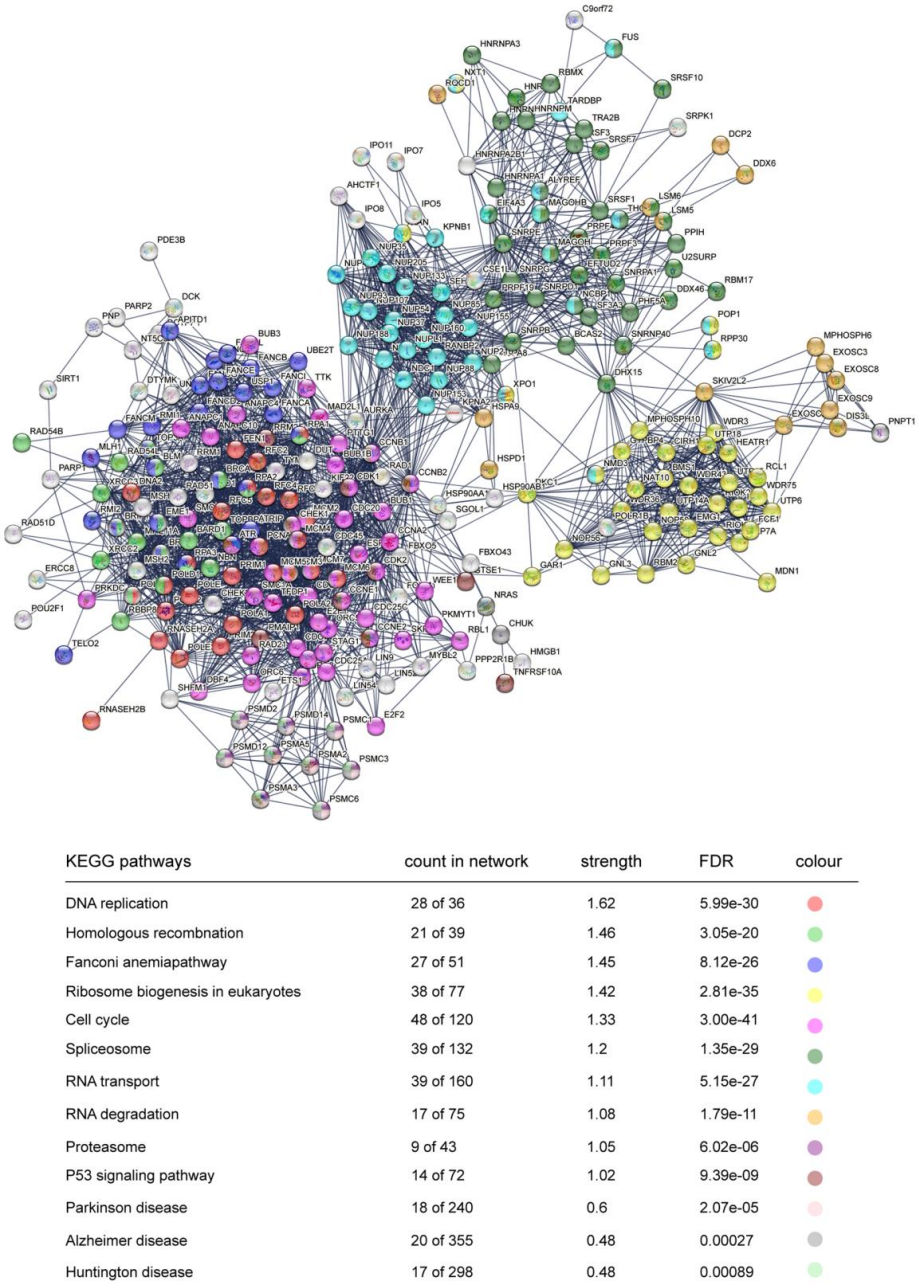
**STRING protein network analysis of downregulated genes.** Interacted proteins are clustered and marked with different colours, representing enriched KEGG pathways.

To better understand the protein–protein interaction (PPI) network of DEGs in the trisomy/DS group, we applied the online interaction database STRING to identify the core network and enriched GO-BP or KEGG pathways acting as regulators in the DS phenotype, especially for the HSA21 genes containing DEGs. After conditional filtering, only interactions with highest confidence of data support could be retained. In the upregulated DEGs, 7 candidate core networks containing HSA21 genes were screened. The top 4 were “Cell matrix adhesion and organization” (*COL6A1*, *COL6A2*, *COL18A1*, *JAM2*, *ADAMTS5* and *POFUT2*), “Immune/virus infection response” (*MX1* and *MX2*), “Histone modification and chromatin remodeling” (*NRIP1*) and “Glycerolipid and lipid metabolism” (*AGPAT3*) (Figure 4). The other three were “Mitochondrial function and ATP synthesis” (*ATP5PF*, *ATP5PO*), “Synaptic vesicle endocytosis” (*ITSN1*, *SYNJ1*) and “Amyloid formation and metabolism” (*APP*) (Supplementary Figure 3). The PPI network of downregulated DEGs was integrated and mainly functioned as a sign of “DNA replication and cell cycle”, such as *Ki67* and *PCNA* (Figure 5). Additionally, RT-qPCR data confirmed the upregulation of key HSA21 genes in DS-derived amniocytes compared with Normal, highlighting the significant role of dosage effect of HSA21 genes as a mediator for gene expression imbalance and function dysregulation in DS (Supplementary Figure 4A).

## DISCUSSION

Down syndrome (DS, OMIM#190685), one of the most common chromosomal abnormalities in liveborn children, can cause multisystemic dysplasia, especially mental retardation. Since the first report by Down in 1866 [22], an increasing number of scientific studies and clinical findings have described the clinical features, involved pathogenic genes and candidate treatment targets, as well as effective prevention and prenatal screening methods [1]. The extra chromosome 21 (HSA21) was introduced to the pathogenesis of DS in 1959 [23], and the dosage effect of HSA21 included genes is the most widely recognized hypothesis due to confident evidence provided from multiomics analysis and the results of overexpressed HSA21 genes on DS phenotypes in cell and animal models [24–30]. To better understand the effect of HSA21 on the alteration of gene expression profile in DS, multiple transcriptome databases have been established, which do help us know what’s up and down in DS [31]. However, the relationship between HSA21 genes and congenital malformations in DS fetal development is still unclear. Here, we obtained RNA-seq data of amniocytes derived from fetuses diagnosed as DS and euploid, aiming to provide more information about the dysregulated genes associated with cellular processes and pathways in developmental phenotype modulation.

As expected, the mRNA expression profile changed significantly in trisomy 21 versus normal group. Interestingly, through functional enrichment of differentially expressed genes (DEGs), we found that upregulated genes were mainly enriched in “extracellular matrix organization and cell adhesion” and “immune system/virus infection regulation” related cellular processes or pathways. The extracellular matrix (ECM) is composed of diverse proteins, such as collagen, fibronectin, and laminin, whose dynamic concentration change and interaction with the environment may play an important role in maintaining muscle morphology and function during embryonic development [32, 33]. Meanwhile, the immune system, especially for interferon signaling, is also essential in pregnancy and fetal development [34, 35]. This may explain why these genes also directly contribute to embryonic development, including “nervous system development”, “heart and skeletal muscle development” and “face morphogenesis”, indicating a close correlation between altered cellular processes and dysplastic phenotypes manifested in DS fetuses. In contrast, downregulated genes principally affect “DNA replication and cell cycle”, which was revealed as a shared phenotype among multiple aneuploids [36]. Indeed, the downregulated genes may result from the upregulated genes, in other words, the upregulated genes disrupted the developmental program and consequently led to defects in cell proliferation in DS, which was confirmed through proliferation assay using EdU staining (Supplementary Figure 4B, 4C).

To determine the HSA21 gene dosage effect, we firstly analyzed the average log2FC distribution of the whole genes on HSA21, which showed an approximately 1.5-fold increase, consistent with other published studies. Afterwards, DEGs in HSA21 were screened and enriched, in which “cell adhesion”, “virus infection regulation” and “synapse function” reappeared, suggesting that HSA21 genes may participate in stimulating the overall cellular process transformation. To identify the key genes involved in DS fetal phenotypes, the STRING online tool was applied to build PPI networks in DEGs, especially for upregulated genes translated proteins. Corresponding to the GO, KEGG and GSEA results, two leading HSA21 genes involved in the network may play a major role in monitoring the DS fetal phenotype. I) “Extracellular matrix organization”, including *COL6A1*, *COL6A2*, *COL18A1*, *ADAMTS5*, *JAM2*, and *POFUT2*. II) “Immune/virus infection regulation”, including *MX1* and *MX2*. Additionally, the *NRIP1* gene, which contains the Pro-X-Asp-Leu-Ser (PXDLS) motif that can bind to BMAL1/CLOCK and regulate circadian rhythmicity [37], interacts with histone deacetylase and functions as “chromatin remodeling” in histone modification. *AGPAT3*, which belongs to the 1-acylglycerol-3-phosphate O-acyltransferase (AGPAT) family, is involved in glycerolipid and glycerophospholipid metabolism [38] and may also control neuronal development [39]. In addition, the ATP synthase subunits *ATP5PF* [40] and *ATP5PO* [41] may regulate mitochondrial function and ATP synthesis. Eventually, *ITSN1*, *SYNJ1* and *APP* could directly result in nervous system dysfunction, which has been demonstrated in some cell or animal models of DS [42–45].

The major limitation of our study is the lack of gene expression (mRNA or protein) validation in the fetus samples, while the candidate genes involved in cellular process alterations have been illustrated in some databases or articles. For example, *COL6A1* and *COL6A2* were highly expressed in DS fetuses, and their overexpression was closely linked with congenital defects in DS [46–48]; *MX1* and *MX2* were highly expressed in DS blood cells and involved in the interferon signaling pathway [12], which is regarded as one of the determined factors in infections and immunodeficiency, aging, and microglial dysfunction in DS [49–52]. Although we could not conclude that the upregulation of HSA21 genes could directly give rise to developmental disorders in DS, which need further evidence in further studies, these candidate genes involved in cellular function activation or inactivation may be considered potential mechanisms and therapeutic targets in DS developmental abnormalities.

## Supplementary Materials

Supplementary Figures

Supplementary Tables 1-2 and 6-7

Supplementary Table 3

Supplementary Table 4

Supplementary Table 5
