# Supplementary material for: Transcriptome research of human amniocytes identifies hub genes associated with developmental dysplasia in down syndrome
Source: Aging (Albany NY). 2023 Dec 12;15(23):14086–108. doi: 10.18632/aging.205291 (PMC10756088; doi:10.18632/aging.205291)
Supplement: Supplementary Figures [file aging-15-205291-s001.pdf]

SUPPLEMENTARY FIGURES

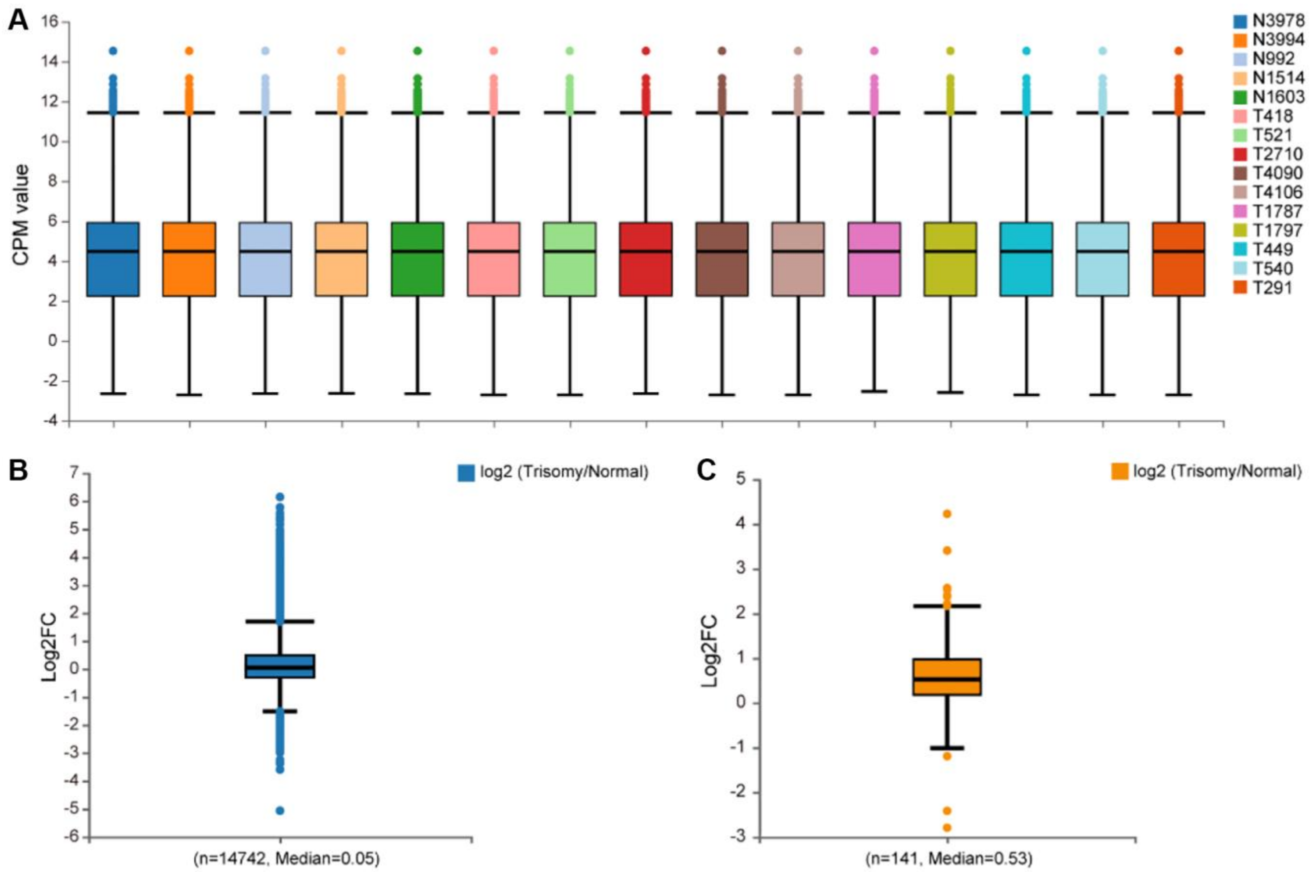

Supplementary Figure 1. Distribution of mRNA expression and fold change in Normal versus Trisomy. (**A**) Boxplot of mRNA expression in each sample from this study. (**B**) Boxplot of all the mRNA fold change distribution in Normal versus Trisomy. (**C**) Boxplot of HSA21 genes' fold change distribution in Normal versus Trisomy.

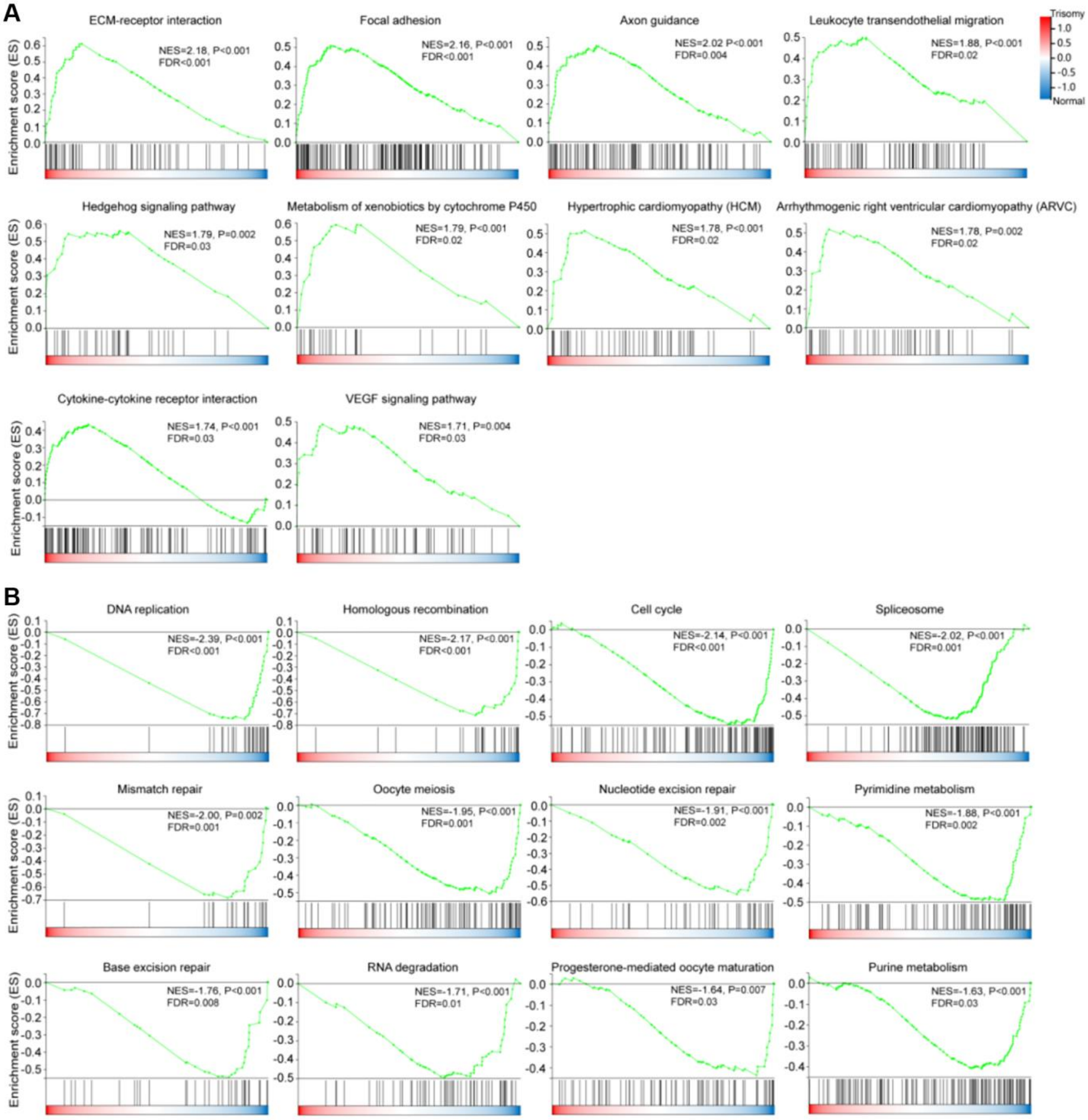

Supplementary Figure 2. Gene set enrichment analysis (GSEA) of all expressed genes in the "C2: curated gene sets" KEGG gene sets collection of MSigDB (Broad Institute). (**A**) Enriched GSEA KEGG pathways in Trisomy group. (**B**) Enriched GSEA KEGG pathways in Normal group.

A

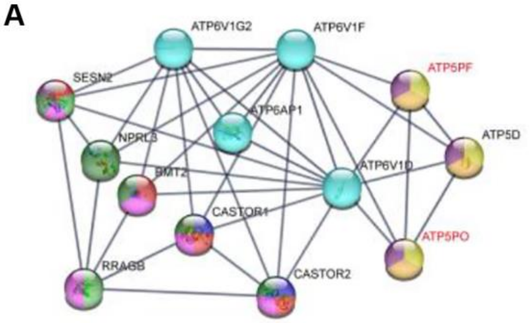

GO-BP term	count in network	strength	FDR	colour
Cellular response to l-arginine	2 of 3	3.0	0.0011	
Negative regulation of torc1 signaling	4 of 17	2.55	1.79e-06	
Cellular response to leucine starvation	2 of 10	2.48	0.0051	
Mitochondrial atp synthesis coupled proton transport	3 of 21	2.33	0.00021	
Regulation of torc1 signaling	5 of 39	2.29	2.47e-07	
Negative regulation of tor signaling	5 of 43	2.24	2.91e-07	
Transferrin transport	4 of 36	2.22	1.50e-05	
Cristae formation	3 of 31	2.16	0.00045	
Mitochondrion organization	4 of 452	1.12	0.0262	

B

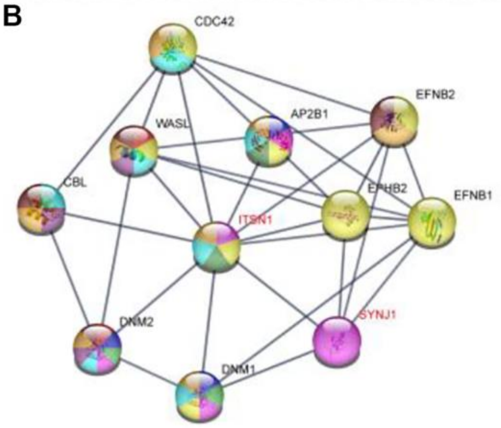

GO-BP term	count in network	strength	FDR	colour
Positive regulation of clathrin-dependent endocytosis	2 of 4	2.95	0.00088	
Postsynaptic neurotransmitter receptor internalization	3 of 8	2.82	1.47e-05	
Synaptic vesicle budding from presynaptic endocytic zone membrane	2 of 7	2.71	0.0017	
Synaptic vesicle endocytosis	5 of 48	2.27	1.00e-07	
Ephrin receptor signaling pathway	8 of 86	2.22	4.36e-13	
Clathrin-dependent endocytosis	2 of 29	2.09	0.0135	
Positive regulation of endocytosis	7 of 103	2.08	2.02e-10	
Positive regulation of receptor-mediated endocytosis	3 of 53	2.0	0.00078	
Regulation of endocytosis	8 of 205	1.84	1.77e-10	
Regulation of receptor-mediated endocytosis	4 of 102	1.84	0.00011	

C

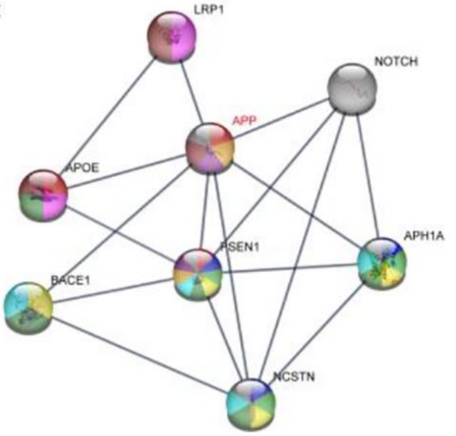

GO-BP term	count in network	strength	FDR	colour
Positive regulation of amyloid fibril formation	3 of 4	3.26	2.58e-06	
Notch receptor processing, ligand-dependent	3 of 7	3.02	4.15e-06	
Amyloid-beta formation	3 of 7	3.02	4.15e-06	
Amyloid precursor protein catabolic process	4 of 12	2.91	8.91e-08	
Positive regulation of amyloid-beta clearance	2 of 7	2.84	0.00088	
Amyloid precursor protein metabolic process	5 of 19	2.81	1.27e-09	
Amyloid-beta metabolic process	4 of 18	2.74	2.68e-07	
Modulation of age-related behavioral decline	2 of 9	2.74	0.0012	
Neuron projection maintenance	2 of 10	2.69	0.0013	
Regulation of amyloid-beta clearance	3 of 17	2.64	2.34e-05	
Notch signaling pathway	5 of 120	2.01	1.16e-06	

Supplementary Figure 3. HSA21 up-regulated genes involved STRING protein network. (**A**–**C**) Secondary 3 networks clustered in the upregulated genes. The HSA21-related genes are marked in red.

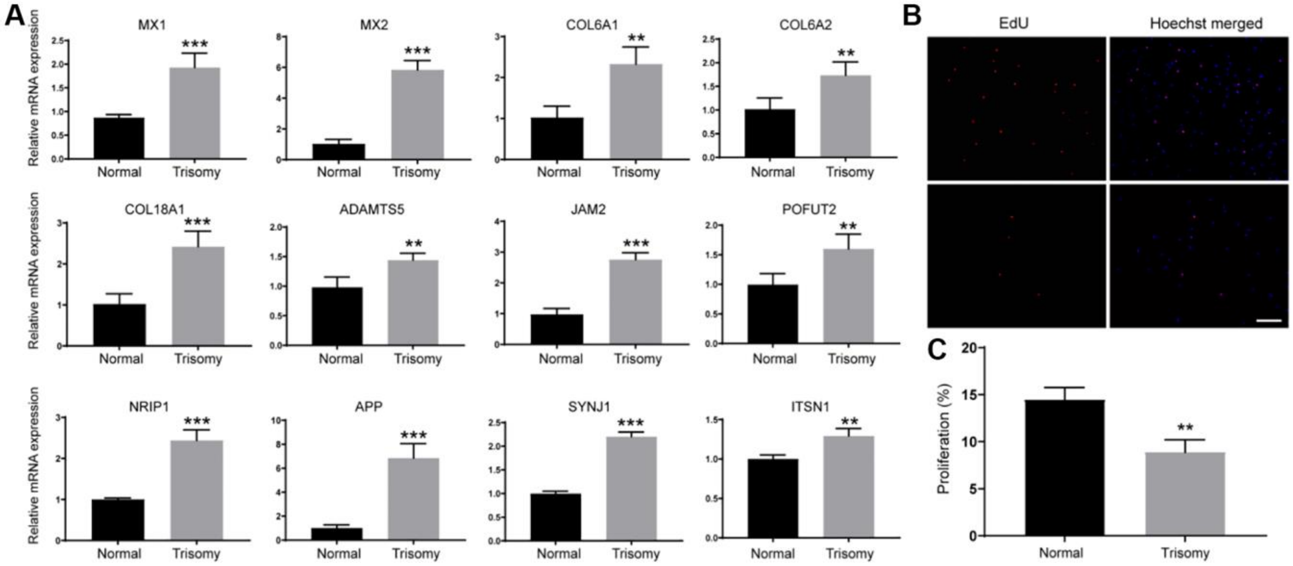

Supplementary Figure 4. Gene expression validation and cell proliferation assay. (**A**) RT-qPCR validation of 12 HSA21 genes (*n* = 4, $^{**}p < 0.01$, $^{***}p < 0.001$). (**B**) Microscope images of EdU staining in amniocytes from Normal and Trisomy group (scale bar = 100 μm). (**C**) Quantitative results of proliferation ability in each group (*n* = 3, $^{**}p < 0.01$).
