# Supplementary material for: Transcriptome research of human amniocytes identifies hub genes associated with developmental dysplasia in down syndrome
Source: Aging (Albany NY). 2023 Dec 12;15(23):14086–108. doi: 10.18632/aging.205291 (PMC10756088; doi:10.18632/aging.205291)
Supplement: Supplementary Tables 1-2 and 6-7 [file aging-15-205291-s002.pdf]

SUPPLEMENTARY TABLES

Supplementary Table 1. Primer sequences used for RT-qPCR.

Gene	Forward primer (5′–3′)	Reverse primer (5′–3′)
**MX1**	GAGCTGGTAAAACGAGGCTG	ATCATTCTCCAAACCTGCGC
**MX2**	AGGGAGAATGAGACCCGTTT	TCCTTGCCTCGATACTGCTT
**NRIP1**	GCCAGAAGATGCACACTTGA	CAAGCTCTGAGCCTCTGCTT
**COL6A1**	CTACACCGACTGCGCTATCA	TCGGTCACCACAATCAGGTA
**COL6A2**	TGGATTCAGCTACCCAGGAC	CTCCTTTCAAGCCAAAGTCG
**COL18A1**	AGAAAGGAGCCAAGGGAGAG	TCCTTTCAATCCGTTCATCC
**JAM2**	TCTGTTGGATATCGCAGGTG	TAGCATACACCAAGGCCACA
**POFUT2**	GAAAGAAGGGACCTGGGAAG	TCCTCATAACCCCAAAACCA
**ADAMTS5**	CTTCACTGTGGCTCACGAAA	TTTGGACCAGGGCTTAGATG
**ITSN1**	ATGGCTCAGTTTCCAACACC	GATCATGCTTCGCTCTTTCC
**SYNJ1**	GGGCGTCTGTTTGTTTGTTT	GGCTACAGACGAAGCAAAGG
**APP**	GAGACACCTGGGGATGAGAA	CTTGACGTTCTGCCTCTTCC

Supplementary Table 2. Sequencing data filtering and mapping to the Homo sapiens (GRCh38) reference genome. "N" means normal, "T" means Trisomy.

Sample Name	Gender	Total raw reads (M)	Total clean reads (M)	Clean reads Q20 (%)	Clean reads Q30 (%)	Clean reads Ratio (%)	Total mapping genome ratio (%)	Uniquely mapping genome ratio (%)
N3978	Male	23.75	23.73	97.31	90.94	99.94	93.79	82.07
N3994	Male	23.63	23.62	97.33	91.04	99.94	93.62	81.77
N992	Male	21.84	21.82	97.36	91.15	99.94	93.73	81.77
N1514	Female	23.92	23.76	97.68	93.2	99.32	95.4	81.7
N1603	Female	23.92	23.76	97.78	93.38	99.32	95.62	82.79
T521	Male	23.92	23.7	97.16	92.36	99.06	94.22	80.7
T418	Male	23.92	23.83	97.82	93.93	99.61	95.78	82.94
T2710	Male	23.92	23.79	97.7	93.12	99.43	95.54	83.95
T4090	Male	23.92	23.76	97.73	93.26	99.33	95.58	82.74
T4106	Male	23.92	23.71	97.78	93.42	99.12	95.5	83.85
T1787	Female	23.92	23.73	98.13	94.38	99.19	95.92	84.22
T1797	Female	23.92	23.78	97.95	93.88	99.41	95.79	84.3
T449	Female	23.92	23.83	97.95	94.29	99.6	95.85	82.99
T540	Female	23.92	23.7	97.25	92.63	99.07	94.29	80.56
T291	Female	23.92	23.79	97.83	94.02	99.46	95.64	81.15

Supplementary Table 6. Enriched regulatory miRNAs which might regulate gene expression in Trisomy group.

NAME	msigdb_c3_mir_mir_ legacy_term_desc	Size (after restricting to dataset)	Original size	ES	NES	NOM *p*-val	FDR *q*-val	Rank at max	Leading edge
TCCAGAG_ MIR518C	Genes having at least one occurence of the motif TCCAGAG in their 3' untranslated region. The motif represents putative target (that is, seed match) of human	127	145	0.52245	2.132416	0	0	2184	tags=32%, list=17%, signal=38%

	mature miRNA hsa-miR-518c* (v7.1 miRBase).								
CCAGGGG_MIR331	Genes having at least one occurence of the motif CCAGGGG in their 3' untranslated region. The motif represents putative target (that is, seed match) of human mature miRNA hsa-miR-331 (v7.1 miRBase).	81	95	0.508043	1.908009	0	0.008027	3059	tags=48%, list=23%, signal=63%
GGGGCCC_MIR296	Genes having at least one occurence of the motif GGGGCCC in their 3' untranslated region. The motif represents putative target (that is, seed match) of human mature miRNA hsa-miR-296 (v7.1 miRBase).	57	73	0.530976	1.862141	0	0.009899	2712	tags=47%, list=21%, signal=60%
GGGACCA_MIR133A_MIR133B	Genes having at least one occurence of the motif GGGACCA in their 3' untranslated region. The motif represents putative target (that is, seed match) of human mature miRNAs hsa-miR-133a and hsa-miR-133b (v7.1 miRBase).	180	201	0.422789	1.804593	0	0.016895	2545	tags=29%, list=20%, signal=36%
TTCCGTT_MIR191	Genes having at least one occurence of the motif TTCCGTT in their 3' untranslated region. The motif represents putative target (that is, seed match) of human mature miRNA hsa-miR-191 (v7.1 miRBase).	27	29	0.585871	1.792728	0.002198	0.017444	1307	tags=33%, list=10%, signal=37%
TTGGGAG_MIR150	Genes having at least one occurence of the motif TTGGGAG in their 3' untranslated region. The motif represents putative target (that is, seed match) of human mature miRNA hsa-miR-150 (v7.1 miRBase).	84	90	0.46768	1.776212	0	0.017839	2071	tags=25%, list=16%, signal=30%
TCTCTCC_MIR185	Genes having at least one occurence of the motif TCTCTCC in their 3' untranslated region. The motif represents putative target (that is, seed match) of human mature miRNA hsa-miR-185 (v7.1 miRBase).	92	124	0.460775	1.755894	0	0.01902	2647	tags=32%, list=20%, signal=39%
GAGCCTG_MIR484	Genes having at least one occurence of the motif GAGCCTG in their 3' untranslated region. The motif represents putative target (that is, seed match) of human mature miRNA hsa-miR-484 (v7.1 miRBase).	87	104	0.456199	1.74654	0	0.021267	3239	tags=38%, list=25%, signal=50%
GAGCTGG_MIR337	Genes having at least one occurence of the motif GAGCTGG in their 3' untranslated region. The motif represents putative target (that is, seed match) of human	141	160	0.42081	1.720128	0	0.024449	3035	tags=38%, list=23%, signal=48%

	mature miRNA hsa-miR-337 (v7.1 miRBase).								
CCCACAT_ MIR2993P	Genes having at least one occurence of the motif CCCACAT in their 3' untranslated region. The motif represents putative target (that is, seed match) of human mature miRNA hsa-miR-299-3p (v7.1 miRBase).	48	53	0.499246	1.707622	0	0.025486	3657	tags=50%, list=28%, signal=69%
GACAGGG_ MIR339	Genes having at least one occurence of the motif GACAGGG in their 3' untranslated region. The motif represents putative target (that is, seed match) of human mature miRNA hsa-miR-339 (v7.1 miRBase).	54	67	0.481195	1.678705	0.008734	0.030485	2980	tags=39%, list=23%, signal=50%
TCCCCAC_ MIR491	Genes having at least one occurence of the motif TCCCCAC in their 3' untranslated region. The motif represents putative target (that is, seed match) of human mature miRNA hsa-miR-491 (v7.1 miRBase).	54	60	0.476821	1.66031	0	0.034482	2968	tags=41%, list=23%, signal=53%
GTGCCAA_ MIR96	Genes having at least one occurence of the motif GTGCCAA in their 3' untranslated region. The motif represents putative target (that is, seed match) of human mature miRNA hsa-miR-96 (v7.1 miRBase).	253	304	0.368728	1.632026	0	0.044122	1686	tags=17%, list=13%, signal=19%

Supplementary Table 7. Differentially expressed genes on HSA21.

Gene symbol	Full name	log2 (FC)	*P*value	*Q*value
GATD3B	Glutamine amidotransferase class 1 domain containing 3B	2.044072127	2.81E-06	6.07E-05
SIK1B	salt inducible kinase 1B	1.18341546	3.66E-04	0.002686298
ADARB1	adenosine deaminase RNA specific B1	1.205730836	6.49E-06	1.17E-04
WDR4	WD repeat domain 4	−0.828616592	4.01E-04	0.00288081
HSF2BP	heat shock transcription factor 2 binding protein	1.554568088	3.87E-07	1.36E-05
ADAMTS5	ADAM metallopeptidase with thrombospondin type 1 motif 5	1.494856859	1.65E-07	7.13E-06
COL6A1	collagen type VI alpha 1 chain	2.405796299	1.03E-15	1.92E-12
COL6A2	collagen type VI alpha 2 chain	2.538711535	9.15E-11	1.87E-08
CSTB	cystatin B	0.848378824	8.78E-08	4.40E-06
CXADR	CXADR Ig-like cell adhesion molecule	0.904156319	0.001373754	0.007497903
RCAN1	regulator of calcineurin 1	0.799566386	0.010044804	0.035748108
POFUT2	protein O-fucosyltransferase 2	1.202986389	4.64E-06	9.1E-04
ICOSLG	inducible T cell costimulator ligand	1.555339436	4.80E-05	5.42E-04
CLDN14	claudin 14	2.178118778	8.40E-05	8.44E-04
USP25	ubiquitin specific peptidase 25	0.675280692	1.16E-07	5.39E-06
HLCS	holocarboxylase synthetase	0.663546966	0.003451792	0.015483813
PRMT2	protein arginine methyltransferase 2	0.858043744	4.14E-15	6.18E-12
IFNGR2	interferon gamma receptor 2	0.961551152	2.03E-13	1.45E-10

APP	amyloid beta precursor protein	1.064880765	3.05E-08	2.02E-06
IL10RB	interleukin 10 receptor subunit beta	0.940053243	6.67E-10	9.31E-08
LSS	lanosterol synthase	0.76232269	1.71E-05	2.47E-04
MX1	MX dynamin like GTPase 1	2.38236566	5.89E-05	6.36E-04
MX2	MX dynamin like GTPase 2	3.409728684	3.03E-04	0.002315045
NCAM2	neural cell adhesion molecule 2	2.571356425	2.12E-04	0.001729262
NDUFV3	NADH:ubiquinone oxidoreductase subunit V3	0.69376096	3.45E-06	7.20E-05
PIGP	phosphatidylinositol glycan anchor biosynthesis class P	1.060262717	8.48E-05	8.48E-04
PFKL	phosphofructokinase, liver type	1.161556614	1.38E-09	1.65E-07
ATP5PF	ATP synthase peripheral stalk subunit F6	0.775627977	2.56E-05	3.32E-04
ATP5PO	ATP synthase peripheral stalk subunit OSCP	0.607605947	3.67E-05	4.39E-04
SLC37A1	solute carrier family 37 member 1	0.796543997	8.87E-04	0.005314895
YBEY	ybeY metalloendoribonuclease	0.807321852	9.50E-07	2.72E-05
CLIC6	chloride intracellular channel 6	−1.188893687	0.013363273	0.044642897
DNAJC28	DnaJ heat shock protein family (Hsp40) member C28	1.03247852	0.003655606	0.016217814
AGPAT3	1-acylglycerol-3-phosphate O-acyltransferase 3	0.755542626	2.36E-09	2.55E-07
MAP3K7CL	MAP3K7 C-terminal like	2.174940448	1.45E-05	2.17E-04
BACH1	BTB domain and CNC homolog 1	0.760421541	0.003499126	0.015625733
JAM2	junctional adhesion molecule 2	1.062419506	0.002731337	0.012890977
EVA1C	eva-1 homolog C	1.570067836	3.79E-04	0.002763159
ITSN1	intersectin 1	0.868284327	4.61E-10	6.82E-08
MRPS6	mitochondrial ribosomal protein S6	1.248989197	8.04E-06	1.40E-04
TMPRSS2	transmembrane serine protease 2	4.232191202	4.43E-05	5.09E-04
TTC3	tetratricopeptide repeat domain 3	0.986379526	4.77E-11	1.11E-08
CFAP410	cilia and flagella associated protein 410	1.461576147	4.23E-08	2.56E-06
TMEM50B	transmembrane protein 50B	0.707728774	0.003073185	0.01413362
COL18A1	collagen type XVIII alpha 1 chain	1.24220949	2.23E-06	5.09E-05
LRRC3	leucine rich repeat containing 3	1.226185586	0.003436494	0.015433252
NRIP1	nuclear receptor interacting protein 1	0.751780285	9.60E-04	0.005639749
SPATC1L	spermatogenesis and centriole associated 1 like	1.394378994	6.50E-10	9.16E-08
PDXK	pyridoxal kinase	0.732069197	8.14E-06	1.41E-04
CBS	cystathionine beta-synthase	1.162119708	3.61E-05	4.32E-04
SYNJ1	synaptojanin 1	0.652982456	7.33E-05	7.54E-04
